# The impact of ribosome association on lncRNA stability: a new layer of post-transcriptional control?

**DOI:** 10.1042/BST20253024

**Published:** 2025-09-12

**Authors:** Courteney K. Pienaar, Benjamin P. Towler, Sarah F. Newbury

**Affiliations:** 1 School of Life Sciences, University of Sussex, Brighton, U.K; 2 Brighton and Sussex Medical School, University of Sussex, Brighton, U.K

**Keywords:** long non-coding RNA, nonsense-mediated decay, RNA stability, RNA turnover, smORFs, translation

## Abstract

Long non-coding RNAs (lncRNAs) play crucial roles in cellular processes; however, the mechanisms controlling their stability are not well understood. Since the appropriate levels of lncRNAs in cells are required to carry out their functions, it is critical that their degradation is tightly controlled. Extensive research has shown that translation and degradation of messenger RNAs (mRNAs) are intricately linked, with repression of translation usually leading to degradation of the RNA. Recently, evidence has emerged to suggest that translation may also affect lncRNA stability. Ribosome engagement may stabilise lncRNAs by protecting them from nucleases or by promoting their degradation via ribosome-associated decay pathways such as nonsense-mediated decay. In this review, we first highlight specific human diseases that result from misregulation of lncRNA stability. We then explore the mechanisms underlying ribosome association and lncRNA stability, drawing comparisons with canonical mRNA mechanisms and highlighting emerging hypotheses that may be particularly relevant to lncRNAs. We also discuss how advanced techniques such as ribosome profiling can be applied to investigate whether lncRNAs are translated. Finally, we suggest future strategies to aid further understanding of lncRNA stability and its relationship with development and disease. Understanding the dynamic relationship between translation and lncRNA decay offers broad implications for RNA biology and provides new insights into the regulation of lncRNAs in both cellular and disease contexts.

## Introduction

Controlling RNA stability is essential for regulating gene expression and maintaining cellular homeostasis. RNA stability determines how long a transcript is available in the cell to perform its function and is a key mechanism of post-transcriptional gene regulation [1]. Previous studies have estimated that up to 50% of changes in total polyadenylated (polyA) messenger RNA (mRNA) levels can be attributed to the regulation of mRNA stability, highlighting its key role in shaping gene expression profiles [2]. Understanding RNA turnover is therefore critical for understanding how and why specific RNAs are differentially regulated.

To date, research in RNA stability has primarily focused on protein-coding RNAs (mRNAs). However, advances in high-throughput transcriptomic technologies have led to the discovery of thousands of long non-coding RNAs (lncRNAs), which are now emerging as a significant focus of study. lncRNAs are a diverse group of transcripts, classically defined as RNA transcripts longer than 200 nucleotides, which lack a significant open reading frame (>100 amino acids in length) [3]. They are transcribed by RNA polymerase II, often display tissue-specific expression patterns, and have well-characterised roles in various molecular processes, including chromatin remodelling, transcriptional regulation and post-transcriptional control [4–6].

Interestingly, ribosome profiling studies have revealed that many lncRNAs are associated with ribosomes. For example, up to 70% of cytosolic lncRNAs interact with ribosomes in the human leukaemic K562 cell line [7]. It has also been shown, in a variety of cell lines and tissues, that a subset of lncRNA transcripts contain small open reading frames (smORFs) and some have even been shown to encode conserved biologically functional peptides [8–10]. For example, in human cardiac muscle cells, the lncRNA *LOC100507537* contains a conserved open reading frame DWORF (dwarf open reading frame), encoding a 34-amino acid micropeptide. The functional micropeptide enhances muscle contractility by localising to the sarcoplasmic reticulum membrane and activating the sarcoendoplasmic reticulum calcium ATPase (SERCA) pump [11]. Similarly, in *Drosophila melanogaster,* the lncRNA *sarcolamban* encodes two micropeptides essential for regulating cardiac muscle contraction [10]. These findings suggest that the proteome may be more complex than previously thought and challenge the traditional definition of lncRNAs, indicating that these transcripts may have previously unrecognised functions. Furthermore, in the context of RNA stability, ribosome association introduces an additional layer of regulation e.g. nonsense-mediated decay (NMD) [12,13]. Despite this emerging evidence, the role of ribosome association in lncRNA stability has not been comprehensively reviewed.

This review examines the interplay between ribosome association and lncRNA turnover, comparing these mechanisms to canonical mRNA turnover pathways and highlighting evidence that the ribosome–lncRNA axis plays a pivotal role in regulating lncRNA stability. We begin by highlighting disease examples associated with lncRNA dysregulation and further discuss translation-associated quality control mechanisms, which are well characterised in mRNAs. We then explore recent advances in molecular techniques that enable the study of lncRNA–ribosome interactions and present studies showing that lncRNAs can undergo active translation. Finally, we summarise evidence linking ribosome association to lncRNA half-life.

## Biological implications and disease relevance of lncRNA stability

The advent of next-generation sequencing revealed the pervasive transcription of thousands of lncRNAs, initially dismissed as biological noise. However, extensive experimental evidence has since established their critical roles in diverse cellular processes, including chromatin modulation, scaffolding of protein complexes, transcriptional regulation, mRNA splicing control and miRNA sequestration [13]. Given these essential functions in normal cellular processes, precise regulation of lncRNA expression and stability is crucial.

Mounting evidence demonstrates that dysregulation of lncRNA stability, whether through inappropriate accumulation or premature decay, contributes to various diseases. For example, nuclear paraspeckle assembly transcript 1 (NEAT1) is an lncRNA that shows increased expression levels (>2-fold) in peripheral blood mononuclear cells and arterial plaques from patients with atherosclerotic cardiovascular disease compared with normal controls. Mechanistically, the RNA editing enzyme ADAR1 (Adenosine deaminase acting on RNA 1) was found to increase A to I editing at specific sites within the *NEAT1* lncRNA, resulting in increased *NEAT1* stability as well as expression levels. ADAR-mediated editing of *NEAT1* appears to facilitate unfolding of a secondary structure within this RNA, allowing the RNA-binding protein AUF1 (AU-rich element RNA-binding protein 1) to bind and stabilise the RNA. Increased levels of *NEAT1* in vascular endothelial cells are associated with increased TNF-α (Tumour necrosis factor 1 alpha) levels, resulting in increased levels of chemokines such as CXCL8 (IL-8 or chemokine (CXC motif) ligand 8). Therefore, control of *NEAT1* stability is important in controlling inflammation during atherosclerosis [14].

The lncRNA *TIALD* (transcript that induces AURKA lysosomal degradation) provides another striking example of how dysregulated lncRNA stability influences disease progression [15]. In hepatocellular carcinoma, *TIALD* levels are significantly reduced compared with normal tissue, with this decreased expression correlating with poor patient survival. The stability of *TIALD* is controlled through m6A (N6-methyladenosine) methylation by METTL16, which acts to increase RNA stability, thereby affecting the degradation rate of *TIALD*. When *TIALD* is stabilised, it suppresses cancer progression by promoting the degradation of Aurora kinase A (AURKA) through the lysosomal pathway, which inhibits tumour cell migration and invasion. This regulatory axis demonstrates how precise control of lncRNA stability is crucial for maintaining normal cellular function and how its disruption can contribute to disease progression.

These examples highlight that lncRNA stability regulation represents more than just a cellular housekeeping mechanism: it is a fundamental control point in gene expression with direct implications for human health. By enhancing our understanding of the mechanisms that govern lncRNA degradation, we open new avenues for therapeutic intervention. Developing methods to further investigate and manipulate lncRNA stability and translation could lead to innovative treatments for diseases where they play significant roles.

## The ribosome influences RNA stability

The ribosome’s role extends far beyond its classical function within the central dogma of gene expression, where it was primarily viewed as a decoder of mRNA sequences for polypeptide synthesis. Several studies have since revealed that the ribosome influences mRNA stability through multiple mechanisms. During translation, ribosomes can protect mRNA transcripts from degradation by physically blocking ribonuclease access, as demonstrated across various organisms. For instance, in *Escherichia coli*, ribosomes physically inhibit RNase E cleavage of the *rpsO* mRNA (encoding ribosomal protein S15), protecting it from downstream exonucleolytic degradation [16]. Similarly, in *Drosophila*, the repression of translation of the maternal mRNA *nanos* leads to its decay, highlighting how the physical presence of ribosomes on transcripts directly influences their stability [17].

The relationship between translation efficiency and mRNA stability is partly explained by the mechanism of codon optimality [18–21]. More efficiently translated mRNAs generally show greater resistance to degradation, with optimal codons promoting stable translation elongation, while suboptimal codons slow ribosome transit. In yeast, the Ccr4/Not (Carbon Catabolite Repression 4—Negative On TATA-less) complex directly interacts with the ribosome to monitor its speed, communicating with the decapping complex to trigger mRNA decay when ribosomes reduce their translation rate [22]. Codon optimality varies between species and tissue types. In humans, codons with a G or C at the third position (GC3) are associated with increased transcript stability, while those with an A or U at the third position (AU3) typically lead to reduced stability [23]. It is thought that ILF2 (Interleukin enhancer-binding factor 2) modulates the stability of low GC3/high AU3 transcripts by binding to AU3-rich regions and triggering their decay [23].

While ribosome engagement can stabilise mRNAs, stalled or aberrant interactions can trigger various decay pathways. A well-studied mechanism of interaction between translation and mRNA degradation is NMD, which targets transcripts containing premature termination codons (PTCs) located ≥50–55 nucleotides upstream of an exon–exon junction, abnormally long (>1 kb) unstructured 3′ untranslated regions (UTRs), upstream open reading frames, or introns within 3′ UTRs [24,25]. Additional pathways include non-stop decay, which eliminates mRNAs lacking stop codons by detecting stalled ribosomes at the 3′ end [26]. These pathways ensure the persistence of only functional transcripts, demonstrating the sophisticated coupling between mRNA decay and translation.

Ribosome collisions occur when trailing ribosomes collide with stalled, leading ribosomes and can also trigger mRNA decay. Ribosome collisions also activate the ribosome quality control and No-go decay (NGD) pathway [26]. These can be detected in polysome profiles; the RNAs protected by collided disomes can also be identified using a modification of the Ribo-Seq protocol below [27]. When ribosomes stall and collide, endothelial differentiation related factor 1 (EDF1) recognises the collision and recruits GIGYF2-4E2 (Grb10-Interacting GYF Protein 2/eukaryotic translation initiation factor 4E-2) [28,29]; meanwhile, ZNF598 (Zinc Finger Protein 598, E3 Ubiquitin Ligase) ubiquitinates ribosomal proteins [30,31]. These events lead to endonucleolytic cleavage by N4BP2 (NEDD4 Binding Protein 2) [32,33], followed by XRN1-mediated degradation and ribosome rescue through the ASC-1 (Asc-type amino acid transporter 1) complex helicase activity [34] or via the PELO/HBS1/ABCE1 (Pelota/HBS1 like translational GTPase/ATP binding cassette subfamily E member 1) pathway [26,35]. Translocation along the RNA transcript can also be important in the regulation of stability. Inhibition of translation elongation using drug treatments such as emetine or anisomycin (which inhibit translocation or disrupt tRNA binding, respectively) stabilises a set of short-lived RNAs in human HEK-293T (Human Embryonic Kidney) cells in a process that is independent of NMD and ribosome collisions. It is possible that the availability of the E-site on elongating ribosomes may be critical for the normal degradation of these RNAs. Similarly, in *Saccharomyces cerevisiae,* short-lived Xrn1-sensitive lncRNAs accumulate in cells during translation inhibition (induced by cycloheximide treatment), suggesting co-translational decay by Xrn1 [36,37].

Recent research has also unveiled the intricate interplay between SKIV2L (Ski2-like RNA Helicase) and AVEN (Apoptosis And Caspase Activation Inhibitor) in monitoring and managing aberrant translation events [38]. AVEN functions by binding to GC-rich, structured RNAs, thereby preventing ribosome stalling either through helicase recruitment or direct melting of G-rich/G-quadruplex structures via its RGG/RG (arginine-glycine-glycine) domain. When AVEN fails to resolve stalling, the helicase SKIV2L, acting as a translation-surveillance factor, is recruited to disome-occupied regions and initiates 3′–5′ decay through the multicomponent exosome complex. These examples demonstrate that the ribosome influences RNA stability, suggesting that translation of lncRNAs may be key in regulating their stability.

## Techniques for studying ribosome–RNA interactions

Experimental evidence for ribosome association with RNAs (including lncRNAs) relies on three key methodologies: **polysome profiling**, **ribosome profiling (Ribo-Seq) and Poly-Ribo-Seq**.

**Figure 1:**
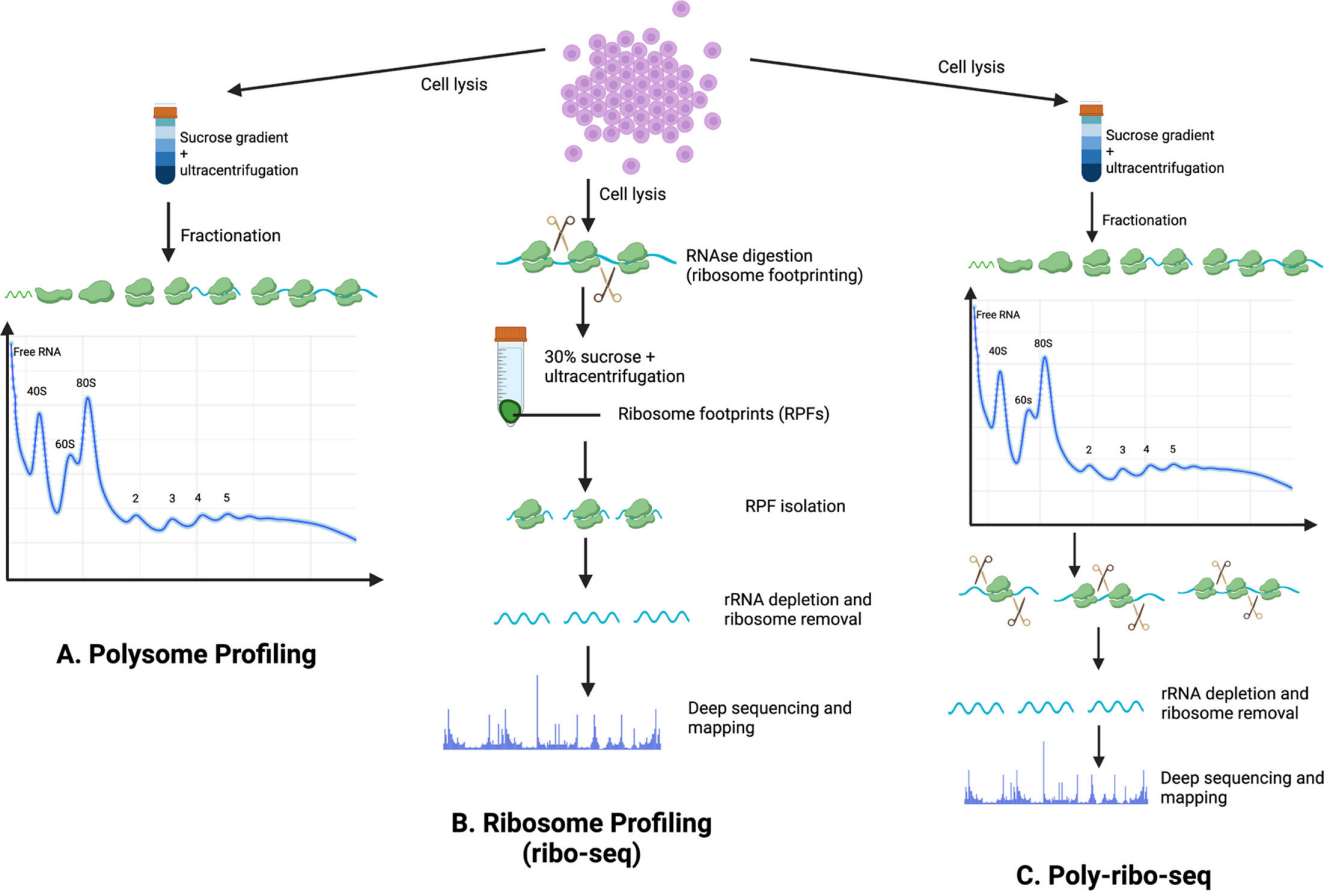
Comparison of techniques for translational profiling. (**A**) Polysome profiling involves the fractionation of mRNA-ribosome complexes through sucrose gradient which separates free RNA, ribosomal subunits (40S and 60S) monosomes (80S) and polysomes. (**B**) In ribosome profiling, cell lysates are subjected to RNase digestion to produce ribosome-protected footprints (RPFs). These footprints are sequenced and mapped to the transcriptome, offering precise positional data and quantitative insights into ribosome occupancy on mRNAs. Framing (Figure 2) of these footprints can

**Polysome profiling** separates cytoplasmic RNAs based on their ribosome association, providing insights into RNA distribution and translational activity [36,39]. This technique involves loading cell lysate onto a linear sucrose gradient, which is then subjected to ultracentrifugation. Following ultracentrifugation, the gradient is passed through a spectrophotometer, which detects and differentiates RNAs based on the number of bound ribosomes to produce a polysome (gradient) profile. Fractions can be collected and processed for use in downstream applications, e.g. quantitative real-time PCR, Western blotting, Northern blotting or sequencing (Figure 1A).

**Figure 2:**
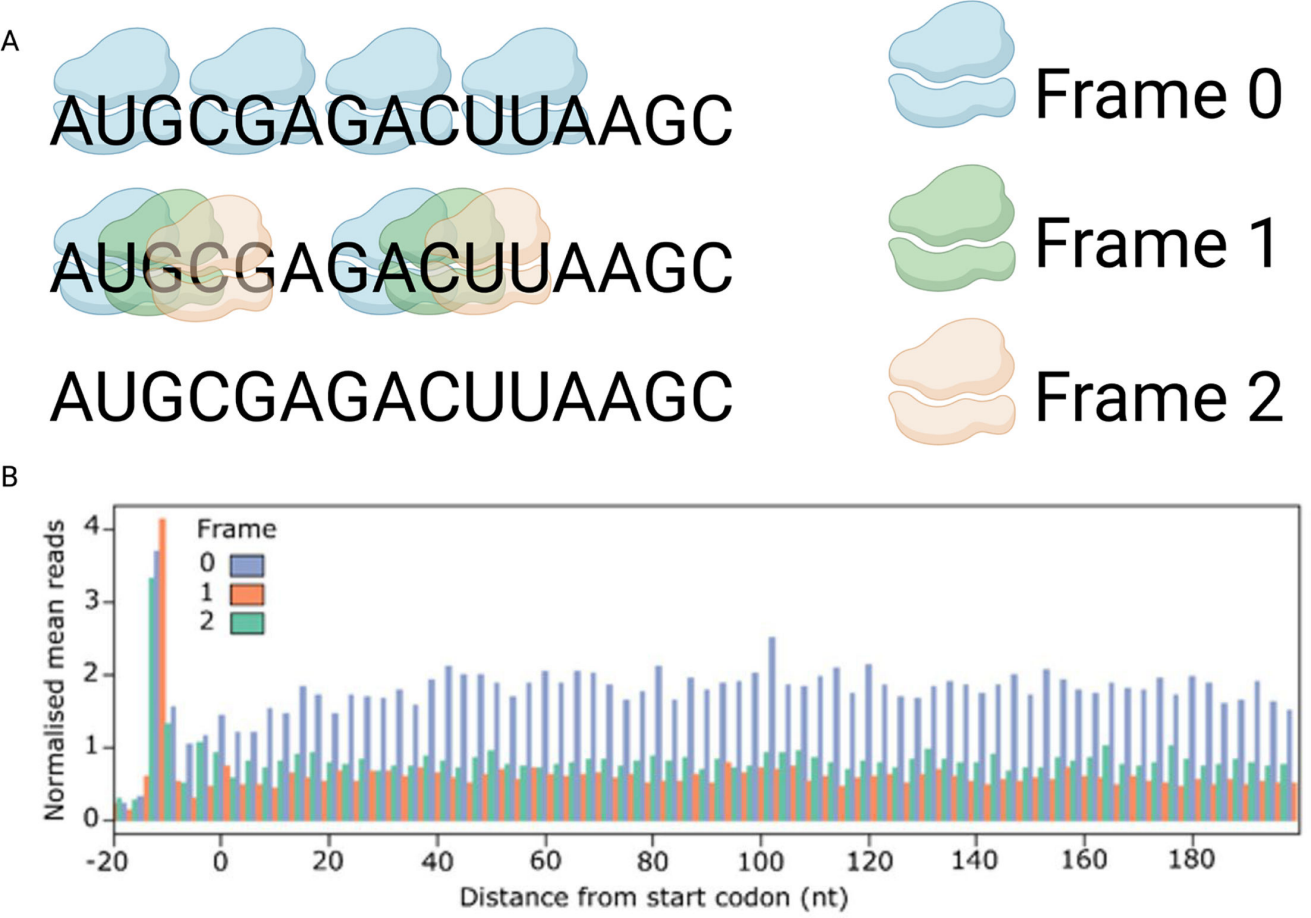
Framing in ribosome profiling (Ribo-Seq) data. In Ribo-Seq experiments, ribosome-protected footprints (RPFs) are analysed to determine whether ribosomes are actively translated and identify the frame in which translation occurs. (**A**) If active translation is occurring, the ribosome will ‘hop’ along the RNA as shown for the ‘blue’ ribosomes translating Frame 0. The ‘green’ and ‘orange’ ribosomes are associated with the RNA but not actively translating. (**B**) Normalised mean reads for each frame as a function of distance from the start codon (nt). Analysis of Riboseq data using Ribotricer shows that there is a strong preference of ribosome occupancy in Frame 0 (blue), which is indicative of high-quality data and reflects active translation. Created in https://BioRender.com

**Ribo-Seq** involves deep sequencing of ribosome-protected RNA fragments (RPFs) and is based on the principle that ribosomes protect 28–32 nucleotide RNA segments from nuclease activity [37,39–45]. Sequencing of these RPFs generates high-resolution maps of ribosome positions across the transcriptome, providing a snapshot of active translation (Figure 1B). Consequently, Ribo-Seq is a useful technique to study lncRNA–ribosome interactions. Provided that samples are sequenced at sufficient depth, this technique allows for the detection and translatomic analysis of even lowly expressed transcripts, including lncRNAs. A critical aspect of Ribo-Seq analysis involves the determination of ribosomal framing, which refers to the reading frame or position in which codons are decoded during translation (Figure 2). Since ribosomes read mRNA nucleotide sequences in triplets, a transcript can theoretically be read in any one of three possible frames; strong preference for a single reading frame within an Ribo-Seq dataset is indicative of high-quality data (Figure 2C) [46]. This framing analysis is essential to differentiate whether a transcript is likely to be actively translated or if the ribosomes (or large protein complexes) are merely associated with the RNA. Through this approach, Ribo-Seq allows for the identification of non-canonical translation events, estimation of translation efficiency and analysis of translational regulatory mechanisms, ultimately providing valuable insights into protein synthesis and gene expression control.

**Poly-Ribo-Seq** is a variant of Ribo-Seq (Figure 1C) [47]. The initial experimental stages are similar to that of polysome profiling whereby cell lysates are loaded onto a sucrose gradient and following ultracentrifugation, the sample is fractionated to separate mRNA transcripts based on the number of ribosomes bound to them. Fractions containing 2+ ribosomes (termed polysomes) are then subjected to nuclease digestion to produce RPFs, like Ribo-Seq, which can then be sequenced to provide information about ribosome density and positionality along the transcript. Poly-Ribo-Seq is particularly useful for analysing lncRNA–ribosome interactions, as it increases confidence that detected translation events are genuine rather than spurious ribosome (or large protein complex) binding. This is especially important when investigating lncRNA–ribosome interactions, where the likelihood that ribosome association represents true translation is lower than for canonical mRNAs. However, the major challenge of performing Poly-Ribo-Seq is that the required input of starting material is much greater than that required for Ribo-Seq, making it difficult to implement when samples are scarce or challenging to harvest. The major advantage of Poly-Ribo-Seq over Ribo-Seq, however, is its ability to reduce false-positive translation events by focusing on polysomes, which are more likely to represent genuine translation events and may minimise contamination from larger cytoplasmic structures.

The above techniques can be combined with other protocols to assess the interplay of translation and stability. For example, performing Ribo-Seq on samples derived from cells or tissues carrying mutations in key ribonucleases, for example Xrn1, can determine whether Xrn1 affects the levels of translated lncRNAs [37]. Alternatively, methods using metabolic labelling, e.g. TLL-seq, can simultaneously assess translation status and levels of transcript in response to the addition of translation inhibitors [36]. The advantages and disadvantages of various techniques to measure RNA turnover are discussed elsewhere [48].

## lncRNAs are often associated with ribosomes and may be translated

Ribo-Seq, along with its derivative technique Poly-Ribo-Seq, has revealed that a subset of lncRNAs exhibit signals consistent with translation events [13,37,40,41,49–51]. For example, a study using Poly-Ribo-Seq, conducted on human neuroblastoma SH-SY5Y cells, revealed that up to 70% of cytoplasmic lncRNAs are associated with polysome complexes [52]. Analysis of multiple Ribo-Seq and RNA-seq datasets across 10 different human cell lines and tissue types (fibroblasts, RPE-1 (retinal pigment epithelial cells), myeloma, ES (embryonic stem cells), HEK-293T, HeLa (cervical cancer cells), PC3 (prostate cancer cells), U2OS (osteosarcoma cells), brain and breast) indicates that 24–59% of expressed lncRNAs are ribosome-associated, with similar findings (26–70%) observed in eight mouse cell lines and various tissues (fibroblasts, EB (embryoid bodies) , ES, brain, hippocampi, skin, liver and testis [53]). These findings, among others, suggest that ribosome association with lncRNAs may not be an anomaly but rather a default destination for many cytoplasmic lncRNAs.

lncRNAs can include more than one ORF, with the main ORF being translated at different times during development. For example, in *Drosophila*, 259 lncRNA ORFs (89% of those translated at any stage) are predicted to show developmental stage-specific translation, while only 11% (33 out of 292) have a constitutive translation signal throughout development. In humans, one study identified 1204 ORFs within 510 lncRNAs, suggesting that human lncRNAs often encode more than one ORF, which could be differentially regulated during developmental stress conditions [54]. It is not easy to experimentally determine whether small ORFs within lncRNAs produce biologically relevant levels of functional peptides. This is because lncRNAs themselves are often present in low abundance, and/or translation of peptides encoded within ORFs may only occur at certain developmental stages and/or under specific stress conditions [52,55,56]. While ‘framing’ (see above and Figure 2) can suggest that ORFs within lncRNAs are translated, experimental validation of peptide expression (e.g. using mass spectrometry, generating fusion peptides and/or measuring a specific phenotype) is still required. Nevertheless, there is a rapidly growing list of biologically functional lncRNA-encoded peptides [40,41,57]. For example, in humans and mice, the lncRNA *MFRL* (mitochondrial function-related lncRNA) encodes a 64-amino acid micropeptide MFRLP [58]. This micropeptide interacts with mitochondrial cytochrome b, where it reduces the accumulation of reactive oxygen species and suppresses mitophagy. By doing so, MFRLP prevents vascular smooth muscle cells from switching from their contractile phenotype, which maintains vascular structure, to a synthetic phenotype associated with arterial remodelling. Another example is the micropeptide SPAR (Small regulatory Polypeptide of Amino Acid Response), encoded by the lncRNA *LINC00961* in both mice and humans. SPAR interacts with lysosomal vacuolar ATPase to negatively regulate mTORC1 (mechanistic target of rapamycin complex 1), a key metabolic signalling pathway [59]. Finally, recent research in myeloma cells carrying mutations in *DIS3* (gene encoding a 3′–5′ exoribonuclease) shows that overexpression of peptides expressed from lncRNAs may contribute to disease progression [60]. If we can control the stability and translation of these transcripts, it might be possible to enhance endogenous peptide production, offering novel strategies for cancer therapy.

## Mechanisms linking translation to lncRNA stability

Given that translation and its associated regulatory mechanisms are well-established modulators of mRNA stability, and considering the growing evidence that cytoplasmic lncRNAs frequently engage with ribosomes and undergo active translation, the lncRNA–ribosome axis likely plays a crucial role in regulating lncRNA stability and fate. While some evidence suggests that the mechanisms linking translation and degradation of lncRNAs parallel those of mRNAs, emerging data highlight interesting differences between the two.

**Figure 3:**
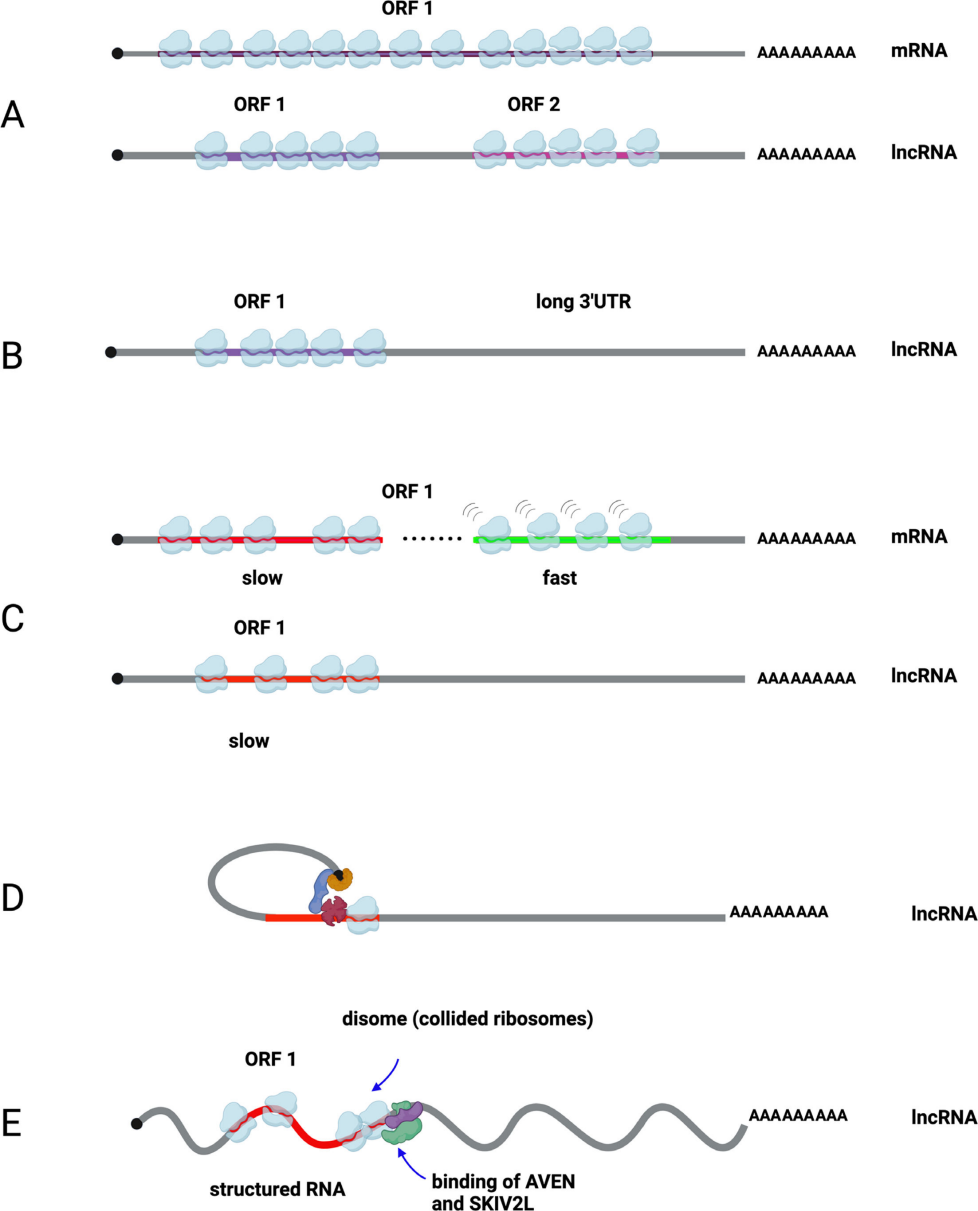
Diagrammatic representation of differences between mRNA and lncRNA that may affect their stability. (**A**) lncRNAs may have one or more short open reading frames. The translation of these ORFs may be developmentally regulated. (**B**) Short ORFs within lncRNAs may have long 3′UTRs, sensitising them to nonsense-mediated RNA decay. (**C**) Longer ORFs found within mRNAs appear to include a 5′ramp (in red; comprising ~200 nt) which is translated less efficiently compared with the remaining ORF (in green). (**D**) lncRNAs often encode ORFs which are shorter than 200 nt therefore are within the size of the 5′ ramp. (**E**) The eIF3 complex (dark red) associated with a ribosome, eIF4G (blue), eIF4E (orange) and the cap until the end of the short ORF is reached. (**F**) Structured lncRNAs with a short ORF often bind AVEN (purple) and SKIV2L (green) which resolve ribosome collisions. Created in https://BioRender.com. lncRNA, long non-coding RNA; mRNA, messenger RNA; ORF, open reading frame.

NMD significantly contributes to lncRNA turnover across eukaryotes (Figure 3B). In *S. cerevisiae*, over 70% of Xrn1-sensitive antisense lncRNAs (XUTs) undergo NMD-mediated degradation [39,61,62] Similarly, in HeLa cells, transcriptome profiling has revealed that, among the top 1000 NMD targets, a significant proportion consists of non-coding transcripts (9% pseudogenes, 6% long intergenic non-coding RNAs and 4% antisense transcripts) [63]. The prevalence of NMD targeting is further supported by *in silico* analysis showing that 40% of spliced, ORF-containing lncRNAs harbour a PTC >50 nt upstream of their last exon [64].

The *GAS5* (Growth Arrest-Specific 5) lncRNA demonstrates the biological significance of NMD-mediated regulation in lncRNA metabolism [65]. *GAS5* is typically targeted for degradation by the UPF1 (Up-frameshift protein 1)-mediated NMD pathway due to the presence of a PTC [66]. Treatment of non-small cell lung cancer (NSCLC) cell lines A549, H1299 and H1975 with ailanthone, a compound derived from *Ailanthus altissima* (commonly known as the tree of heaven), which inhibits UPF1 translation, impedes NMD and stabilises *GAS5*. The resulting accumulation of *GAS5* suppresses ULK1(Unc-51-like kinase 1)-mediated autophagy, which is critical for tumour cell survival, effectively halting NSCLC progression [65].

Other ribosome-associated decay pathways, such as NGD, may also regulate lncRNA stability. NGD can be triggered by several factors, including the presence of GC-rich sequences or structural elements such as stem loops [26]. While direct evidence for NGD targeting lncRNAs for decay is lacking, one study suggested that ribosome-associated lncRNAs (ribo-lncRNAs) contain strong secondary structures and have a higher GC content than no-ribo-lncRNAs [67], which may target them for NGD.

The relationship between ribosome association and lncRNA stability may differ fundamentally from that of canonical mRNAs due to their distinct structural features. LncRNAs typically contain short open reading frames, with human lncRNA ORFs averaging around 45–55 codons (136–166 nt) [68] and yeast lncRNA ORFs averaging 30 codons (90 nt) [37,39,55]. Given that ribosomes occupy approximately 30 nucleotides, these short ORFs can only accommodate a limited number of ribosomes (about three to five) [69]. This limited ribosome occupancy may reduce the protective ‘shielding’ effect that ribosomes confer on mRNA transcripts, potentially making this mechanism less relevant for lncRNAs. Additionally, the stabilising roles of translational efficiency and codon optimality observed in canonical mRNAs may not apply to lncRNAs in the same way. Genome-wide studies in yeast reveal that short ORFs (<200 codons) exhibit lower ribosome density and translational efficiency compared with longer ORFs, a phenomenon linked to slower elongation speeds at the start of ORFs (5′ translation ramp) (Figure 3C) [70]. Another way that short ORFs could affect the translation and degradation of mammalian lncRNAs is that the 5′ cap may be tethered to the 80S ribosome for about 12–60 codons via an interaction between the eIF3 (eukaryotic initiation factor 3) complex and eIF4G (eukaryotic initiation factor 4G), which in turn is bound to eIF4A (eukaryotic initiation factor 4A) and the RNA cap [71,72]. Since lncRNA ORFs are often shorter than 60 codons, this tethering could occur until the stop codon of the short ORF is reached (Figure 3D). Therefore, lncRNAs could be more susceptible to RNA degradation due to reduced translational efficiency of these short ORFs.

The highly structured nature of many lncRNAs, because of functions such as scaffolding protein complexes, also suggests that they may be particularly susceptible to specialised degradation pathways. Analysis of the RNA targets of AVEN proteins (see above and [38]) has shown that AVEN targets hundreds of ‘non-coding’ GC-rich RNAs encoding small ORFs, where ribosome stalling (as detected by disome occupancy) tends to occur (Figure 3E). Therefore, lncRNAs often appear to be particularly susceptible to degradation by the AVEN-SKI2VL pathway.

Codon optimality represents another translation-associated mechanism that may distinctly influence lncRNA stability. Non-optimal codons, particularly AU3 codons in humans, are associated with transcript destabilisation [23]. While this suggests that AU3-rich lncRNAs might be inherently less stable, the effects of codon optimality are context-dependent. For example, in *S. cerevisiae*, the correlation between codon optimality and stability is absent from very short coding regions (<62 codons) but becomes apparent in longer ORFs (>83 codons) [73]. Given the typically shorter length of lncRNA ORFs, they may operate under different regulatory principles. Further studies are needed to fully unravel the complex interplay between ribosome association, translation and lncRNA stability.

## Future directions

Despite recent advances, many questions remain about the functional consequences of ribosome association for lncRNA stability. Future research should focus on investigating the molecular mechanisms that govern lncRNA stability, such as ribosome association, codon usage and decay pathways like NMD, as well as identifying the ribonucleases responsible for their degradation. Additionally, continued efforts are needed to explore the dual roles of lncRNAs as regulatory RNAs and as templates for peptide production. Understanding how the stability of peptide-encoding lncRNAs influences peptide production and their downstream biological effects will be critical. Furthermore, methods to enhance or suppress the translation of these lncRNAs could provide strategies to increase or decrease endogenous peptide production, offering potential therapeutic avenues, particularly for cancer treatment. Since there is evidence that the translation of a particular ORF within a multi-ORF lncRNA may change during development [52,56], it will also be interesting to investigate whether this switch affects the stability of the lncRNA transcript. Finally, the development of high-throughput screening approaches for modulators of lncRNA stability will advance our understanding of post-transcriptional regulation and reveal potential therapeutic applications in diseases where lncRNA stability is dysregulated.

**Perspectives**

- Understanding ribosome-long non-coding RNA (lncRNA) dynamics will provide insights into fundamental RNA biology and identify novel therapeutic strategies. This knowledge could lead to precision medicine approaches to treat diseases driven by lncRNA dysregulation.
- lncRNAs regularly associate with ribosomes and can be translated into biologically functional peptides. This association can target them for degradation, but the exact mechanisms through which lncRNA stability is controlled at the translational level remain unclear.
- Future advances will require advanced tools and techniques to elucidate the mechanisms underpinning ribosome-associated lncRNA stability. It will also be interesting to analyse lncRNA stability, translation and localisation in natural tissues to understand how developmental transitions and cellular stresses can modulate ORF translation as well as lncRNA stability.

## Abbreviations

ABCE1: ATP binding cassette subfamily E member 1
ADAR1: Adenosine deaminase acting on RNA 1
ASC-1: Asc-type amino acid transporter 1
AU3: A or U at the third position
AUF1: AU-rich element RNA-binding protein 1
AURKA: Aurora kinase A
AVEN: Apoptosis And Caspase Activation Inhibitor
CCR4/NOT: Carbon Catabolite Repression 4 - Negative On TATA-less
CXCL8: IL-8 or chemokine (CXC motif) ligand 8
GAS5: Growth Arrest-Specific 5
GC3: G or C at the third position
GIGYF2-4E2: Grb10-Interacting GYF Protein 2/eukaryotic translation initiation factor 4E-2
ILF2: Interleukin enhancer-binding factor 2
MFRL: mitochondrial function-related lncRNA
N4BP2: NEDD4 Binding Protein 2
NEAT1: nuclear paraspeckle assembly transcript 1
NGD: No-go decay
NMD: nonsense-mediated decay
NSCLC: non-small cell lung cancer
PELO/HBS1: Pelota/ HBS1 like translational GTPase
PTC: premature termination codon
RGG/RG: arginine-glycine-glycine motif
RPFs: ribosome-protected RNA fragments
SKIV2L: Ski2-like RNA Helicase
SPAR: Small regulatory Polypeptide of Amino Acid Response
TIALD: transcript that induces AURKA lysosomal degradation
TNF: Tumour necrosis factor
ULK1: Unc-51-like kinase 1
UPF1: Up-frameshift protein 1
UTRs: untranslated regions
XUT: Xrn1-sensitive unstable transcripts
ZNF598: Zinc Finger Protein 598, E3 Ubiquitin Ligase
eIF3: eukaryotic initiation factor 3
eIF4A: Eukaryotic initiation factor 4A
eIF4G: Eukaryotic initiation factor 4G
lncRNA: long non-coding RNA
m6A: N6-methyladenosine
mRNA: messenger RNA
mTORC1: mechanistic target of rapamycin complex 1
polyA: polyadenylated
